# COVID-19 infection and its association with severe malaria & dengue: an epidemiological study from Southern India

**DOI:** 10.1186/s12879-025-11324-4

**Published:** 2025-07-19

**Authors:** Sara J. Ommen, Prasanna Mithra, Rekha T., Nithin Kumar, Ramesh Holla, Naveen Kulal, Mithun Rao, Bhaskaran Unnikrishnan

**Affiliations:** 1 Department of Community Medicine, Kasturba Medical College Mangalore, Manipal Academy of Higher Education, Manipal, India; 2 District Vector Borne Disease Control Programme Office, Dakshina Kannada, Mangalore, Karnataka India

**Keywords:** COVID-19, Severe malaria, Severe dengue, COVID-19 vaccine

## Abstract

**Background:**

Post-Coronavirus Disease-19 (COVID-19) sequelae involve complex biological processes that can alter the progression and clinical outcome of other infectious diseases. However, there is no documented information on the influence of COVID-19 on the development of severe malaria and dengue. Hence, this study was conducted to determine the association between malaria & dengue and previous COVID-19 infection among the adult population of Mangalore Taluk and to describe the socio-demographic and clinical correlates of malaria & dengue.

**Methods:**

This case-control study was conducted among 293 participants who were positive for either malaria or dengue from November 2022 to August 2024. Data were collected using a proforma which contained sections on demographic details, clinical profile and comorbidities, history of COVID-19 infection and COVID-19 vaccination status. The participants were categorised into having severe disease and mild to moderate disease based on operational definitions. The data were analysed using the Statistical Package for Social Sciences (SPSS) version 29. Chi-square test was done, and a *p*-value less than 0.05 was considered statistically significant. Binary Logistic Regression analyses were used and odds ratios were estimated.

**Results:**

A total of 293 participants were included in the study with a mean age of 36.7 (SD:14.8) years. Among them, 23.9% (*n* = 70) had malaria and 76.1% (*n* = 223) had dengue. Out of those who had malaria, 30% (*n* = 21) developed severe illness, whereas among those who had dengue 35.8% (*n* = 80) developed severe dengue. Overall, 58.4% (*n* = 171) were in the age group of > 30 years. In total, 98.1% (*n* = 52) of the participants with dengue fever with a history of COVID-19 infection developed severe dengue, (aOR:55.93 (95% CI:7.17–435.92) *p* < 0.001) compared to those without. Similarly, 85.7% (*n* = 12) of the participants with a history of COVID-19 infection developed severe malaria (aOR: 263.7 (95% CI: 34.9–1987.4) *p* < 0.001) compared to those and these differences were found to be statistically significant. In addition, those participants belonging to lower socio-economic classes had higher chances of developing severe dengue than those belonging to the upper socio-economic class (*p* < 0.001*).

**Conclusion:**

Those with a prior history of COVID-19 infection had higher chances of developing severe malaria and dengue than those without a history of COVID-19 infection.

**Supplementary Information:**

The online version contains supplementary material available at 10.1186/s12879-025-11324-4.

## Background

Vector-borne diseases are human illnesses caused by parasites, viruses and bacteria transmitted by arthropod vectors [1]. Among them, malaria and dengue are highly prevalent across many regions of the world. Human malaria is a vector-borne disease caused by five different *Plasmodium* species, transmitted through the bite of infected female Anopheles mosquitoes [2]. Severe malaria can manifest as cerebral malaria, anaemia, and respiratory distress and it can be fatal if not promptly and effectively managed. The WHO (World Health Organisation) World Malaria Report 2023 reported a global malaria incidence of 249 million cases [3, 4].

Dengue is another vector-borne disease transmitted by *Aedes* mosquitoes. Dengue infection may be asymptomatic or cause undifferentiated febrile illness, classical dengue fever, or haemorrhagic fever, including Dengue shock syndrome. There are an estimated 96 million symptomatic cases and 40,000 deaths every year [5]. 

Sustainable Development Goal (SDG) 3 aims to ensure healthy lives and promote well-being for all. Target 3.3 particularly focuses on combating infectious diseases. It seeks to tackle the epidemics of AIDS, tuberculosis, malaria and neglected tropical diseases (including dengue) and combat hepatitis, water-borne diseases and other communicable diseases [6]. Malaria and dengue are major public health threats in Southeast Asian countries, particularly India, towards achieving the SDGs. The risk of Vector-borne diseases in India has increased in recent years due to rapid urbanisation and improper water storage practices, which have led to the proliferation of mosquito breeding sites [7]. India accounted for approximately 51% of all estimated malaria cases in the WHO South-East Asia Region in the year 2023 [4]. *Plasmodium vivax* and *Plasmodium falciparum* are two species that infect people with malaria most frequently in India. The National Vector Borne Disease Control Programme (NVBDCP), in India, aims to prevent and control vector-borne diseases including malaria and dengue [2]. 

The COVID-19 pandemic also contributed to the magnitude and sequelae of vector-borne diseases [8]. There may be persistent inflammatory responses and other immunopathological mechanisms underlying post-COVID sequelae, which can alter the natural history and clinical outcome of many infectious diseases [9, 10]. However, there is no documented information on the influence or association of COVID-19 on the severity of Malaria and Dengue among people. Hence this study was carried out to seek insight into such possible association between a history of COVID-19 infection and the severity of current episodes of malaria or dengue, in the urban area of Mangalore, in Dakshina Kannada District within the Indian State of Karnataka. Malaria & Dengue have been endemic diseases in Mangalore for over three decades, contributing 85% of malaria cases in the state of Karnataka [11]. 

## Methods

### Study design

This case-control study was conducted in the urban area of Mangalore between 1 st August 2022 and 19th August 2024. among Malaria and Dengue cases undergoing domiciliary treatment and those admitted to tertiary care hospitals affiliated with a Medical College in Mangalore. Data on malaria and dengue cases were acquired from two primary sources. We periodically obtained community-level positive case list from the District Vector Borne Disease Control Programme (DVBDCP) office. Concurrently, lists of hospitalized malaria and dengue patients were collected from the laboratories of the study hospitals. The severe cases of both malaria and dengue were considered cases, and the mild to moderate cases were considered as controls for this study. (Supplementary material 1) Two settings were included to involve both severe and mild to moderate cases of malaria and dengue.

### Sample size

The sample size was calculated using Kelsey’s equation:

$$\mathrm n=\frac{{(Z\beta\:+\:Z_{\alpha/2})}^2\mathrm p(1-\mathrm p)\;(\mathrm r\:+\:1)}{\mathrm r\;{(\mathrm p0-\mathrm p1)}^2}$$

With an assumption of, the proportion of participants with a history of COVID-19 infection among severe malaria or dengue cases as 70%, & mild to moderate malaria or dengue as 50%, 95% confidence interval, 90% power and 1:2 allocation, the minimum sample size was calculated to 288 (96 severe cases and 192 mild to moderate malaria/dengue cases). However, a total sample size of 293 was obtained (101 severe cases and 192 mild to moderate malaria/dengue) during the study period. We followed a sequential (non-random) sampling technique for the recruitment of the study participants.

### Data collection methodology

After obtaining approval from the Institutional Ethics Committee (IEC), required permissions were taken from the Head of the Institute, the respective hospital authorities and NVBDCP office of the study District. The details of malaria and dengue-positive (diagnosed using the dengue NS1 antigen/IgM antibodies test) cases from the community were procured from the district NVBDCP office, from time to time throughout the study duration. Similarly, lists of admitted malaria and dengue patients were obtained from the laboratories attached to the study hospitals.

**Fig. 1:**
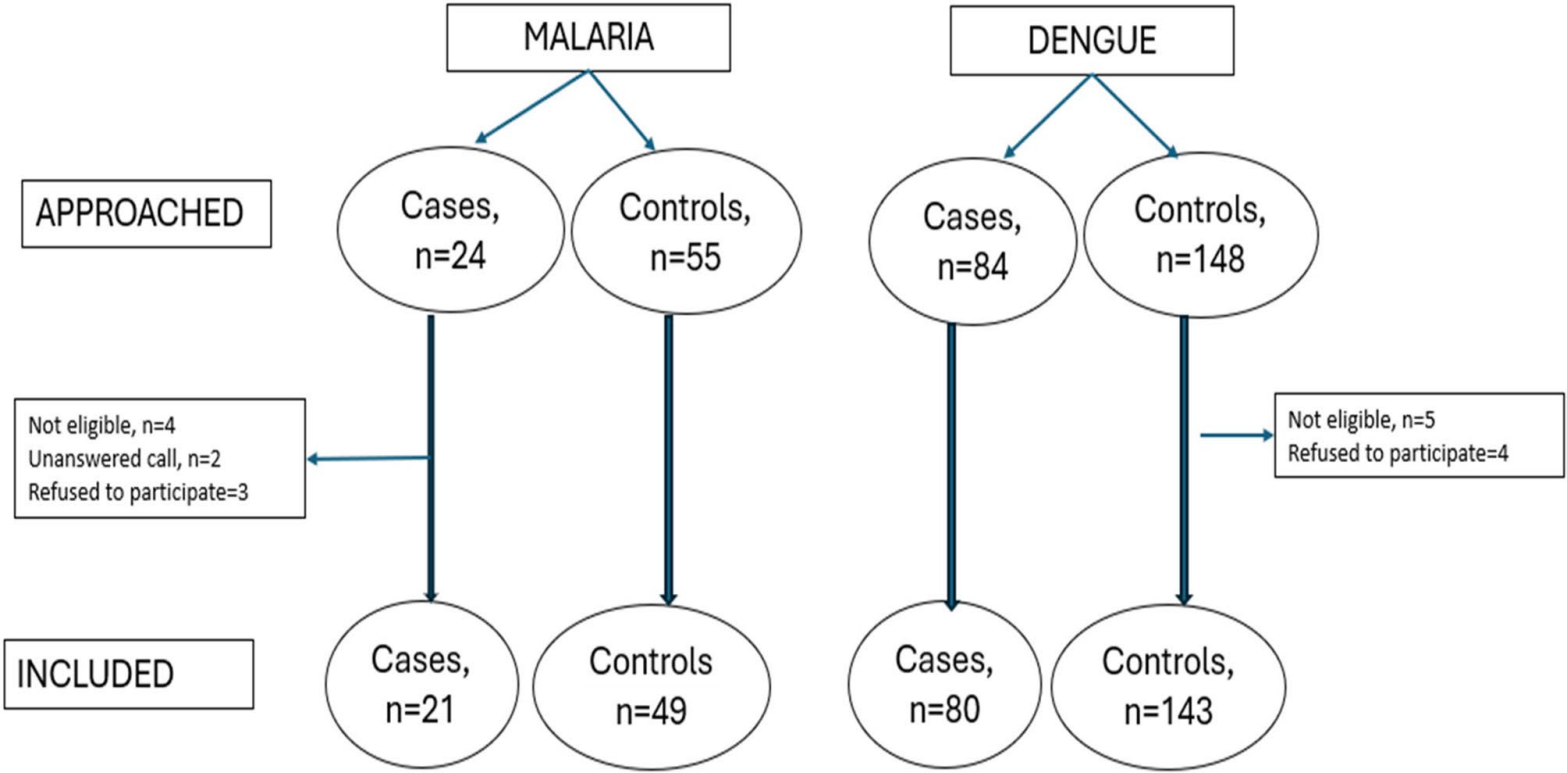
Study recruitment flow diagram

The study hospitals and households of the eligible study participants were visited. Participants were contacted. All adult cases who consented to participate were included in the study (Fig. 1). Those co-infected with malaria and dengue were excluded. The malaria and dengue test reports of the patients were verified. The participants were provided with a participant information sheet and were informed about the objectives of the study in detail in a language understood by them. After obtaining a written informed consent from each one of them, they were recruited into the study. A proforma including personal and family details, clinical details of malaria/dengue, history of previous COVID-19 infection and its severity, hospital admission, medicines consumed, details of any known comorbidities if present, COVID-19 vaccination status, hospitalization and use of any prophylactic medications for COVID-19 was used after validation. COVID-19 positive reports were verified using the confirmation reports of Rapid Antigen Test (RAT) or Reverse Transcriptase PCR (RTPCR) on their mobile phones. This study used the Strengthening the Reporting of Observational Studies in Epidemiology (STROBE) Statement (Supplementary material 2) [12].

### Statistical analysis

The data collected were analysed using IBM SPSS Statistics for Windows, Version 29.0.2.0 Armonk, NY: IBM Corp. results were expressed using proportions and mean (Standard Deviation) The patients were categorized as severe or non-severe Malaria or dengue cases based on the operational definitions (Supplementary material). The factors that were associated with severe malaria/dengue were assessed using the Chi-square test. Bi-variable and Multivariable analysis of the factors determining the severity of malaria/dengue was carried out using Logistic regression. The variance explained by the data was determined using Cox & Snell’s and Nagelkerke’s R^2^ values. The model fit data for the binary logistic regression models were reported. In this model, the confounders that were adjusted were age, gender, education, occupation, socioeconomic status, comorbidities, history of COVID-19 infection and number of COVID-19 vaccine doses taken.

## Results

### General participant information

**Fig. 2:**
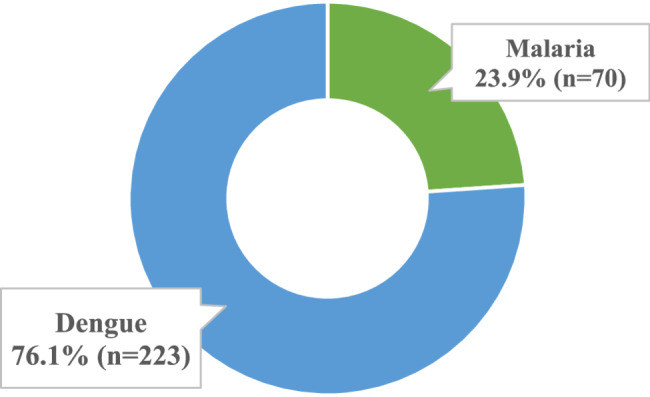
Disease-wise distribution of the study participants (*n*=293)

**Fig. 3:**
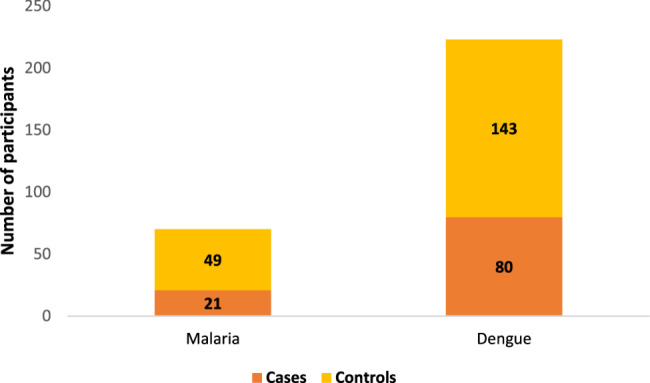
Distribution of the study participants based on severity of disease (*n*=293)

The study recruitment is depicted in Fig. 1. In total 311 eligible participants had to be approached to reach the required sample size. A total of 293 participants were included in the study, out of which 23.9% (*n* = 70) had malaria and 76.1% (*n* = 223) had dengue as depicted in Fig. 2. Out of those who had malaria, 30% (*n* = 21) developed severe illness, whereas among those with dengue, 35.8% had severe dengue disease (Fig. 3). Overall, 48% (*n* = 142) were admitted to the hospitals and 52% (*n* = 151) were undergoing domiciliary management.

### Clinical manifestations & Comorbidity profile

**Fig. 4:**
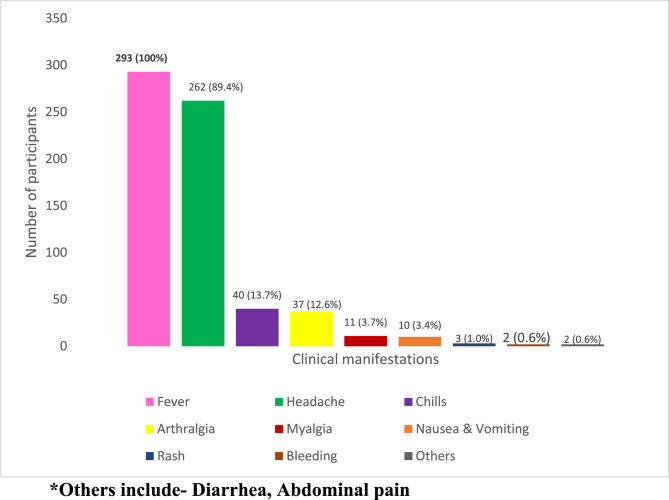
Clinical manifestations of the participants (*n*=293)

**Fig. 5:**
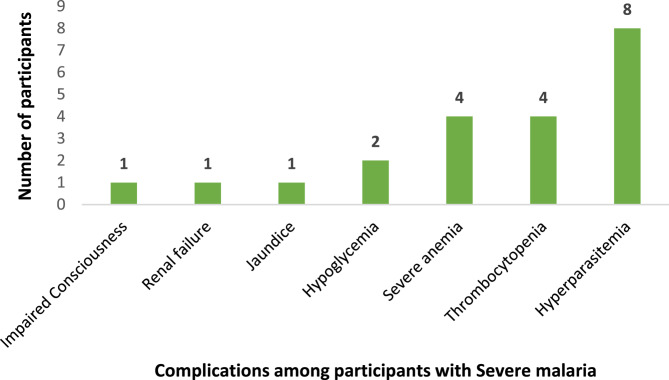
Distribution of complications among patients with Severe Malaria (*n*=21)

**Fig. 6:**
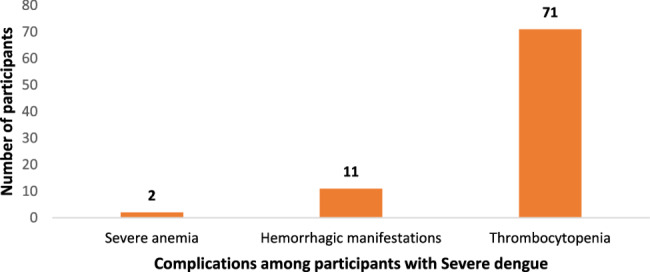
Distribution of complications among patients with severe dengue (*n*=80)

All the participants (*n* = 293) had fever, 89.4% (*n* = 262) had headache, 13.7% (*n* = 40) had chills, as shown in Fig. 4. Participants also manifested chills, arthralgia, myalgia, rashes, and bleeding. It was noted that Hyper parasitemia (*n* = 8) was the most common complication seen among participants with severe malaria (Fig. 5). A higher proportion of those with severe dengue developed thrombocytopenia (*n* = 71) as the complication as shown in Fig. 6.

**Fig. 7:**
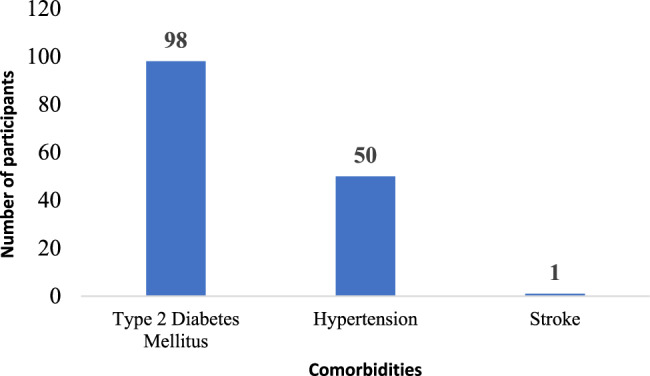
Comorbidity profile as reported by the participants (*n*=293)

The clinical profile of the participants is depicted in Table 1. Among the participants with malaria, 92.9% (*n* = 65) had one or the other comorbidities, 20% (*n* = 14) had history of COVID-19 infection in the past, 4.3% (*n* = 3) had history of previous dengue infection and 12.9% (*n* = 9) had history of previous malaria infection. As shown in Table 2, among those who developed severe malaria, 61.9% (*n* = 13) were infected with Plasmodium falciparum species of malaria. The comorbidity profile of the participants is depicted in Fig. 7. Overall, 33.4% (*n* = 98) reported to be known cases of Type 2 diabetes mellitus, 17.1% (*n* = 50) were hypertensives and one participant (0.003%) had a history of CVA/Stroke in the past.

**Table 1:** Clinical profile of the participants (*n*=293)

**Factors**	**Categories**	**Malaria (*n*, %)**	**Dengue (*n*, %)**
Comorbidities	Yes	065 (92.9)	048 (21.5)
	No	005 (07.1)	175 (78.5)
History of COVID-19 infection in the past	Yes	014 (20.0)	053 (23.8)
	No	056 (80.0)	170 (76.2)
History of previous Dengue infection	Yes	003 (04.3)	001 (00.5)
	No	067 (95.7)	222 (99.5)
History of previous Malaria infection	Yes	009 (12.9)	001 (00.5)
	No	061 (87.1)	222 (99.5)
Type of COVID-19 vaccine	Covishield	062 (88.6)	217 (97.3)
	Covaxin	008 (11.4)	006 (02.7)
No. of doses of COVID vaccine taken	2	045 (64.3)	073 (32.7)
	3	025 (35.7)	150 (67.3)

**Table 2:** Type of malarial parasite & severity of malaria (*n*=70)

**Type of malarial parasite**	**Severe Malaria**	
	**Yes (Cases) *n*, %)**	**No (Controls)** ***n*, %**
Plasmodium vivax	08 (38.1)	44 (89.8)
Plasmodium falciparum	13 (61.9)	05 (10.2)

Among those participants with dengue fever, 21.5% (*n* = 48) had comorbidities, 23.8% (*n* = 53) had history of COVID-19 infection in the past, 1 participant each (0.5%) had history of dengue fever and malaria in the past as shown in Table 3. Also, 64.3% (*n* = 45) of those with malaria had taken < 3 doses of the vaccine and 32.7% (*n* = 73) with dengue had taken < 3 doses of the vaccine. Also, none of the study participants took any type of prophylactic medications for COVID-19. Also, 88.6% (*n* = 62) with malaria and 97.3% (*n* = 217) with dengue had taken Covishield vaccine against COVID-19.

**Table 3:** History of COVID-19 infection and severity of Malaria & Dengue (*N*=293)

		** Severe Malaria (*n*=70)**		***p*-value**^**#**^	**Severe Dengue (*n*=223)**		***p*-value**^**#**^
Historty of COVID-19 infection		Yes (Cases) No. (%)	No (Controls) No. (%)	<0.001*	Yes (Cases) No. (%)	No (Controls) No. (%)	<0.001*
	Yes	012 (85.7)	002 (14.3)		052 (98.1)	001 (01.9)	
	No	009 (16.1)	047 (83.9)		028 (16.5)	142 (83.5)	

### Socio-demographic characteristics

The mean age of the participants was 36.7 (SD:14.8) years with a range of 18–85 years and overall, 58.4% (*n* = 171) were in the age group of > 30 years. In total, 78.5% (*n* = 55) of the participants diagnosed with malaria and 63.7% (*n* = 142) with dengue were males. Among those who had malaria, 70% (*n* = 49) were engaged in unskilled & semiskilled occupation and among those with dengue,72.2% (*n* = 161) were engaged in unskilled & semiskilled work. In our study, socioeconomic status of the participants was determined using the Modified B.G. Prasad Scale 2023 [13]. This scale is widely used in India and is based on per capita monthly family income. According to this scale, 51.4% (*n* = 36) of the participants with malaria, and 71.7% (*n* = 160) with dengue belonged to Socioeconomic class III & below. All the participants received one or the other type of vaccination against COVID-19 infection. Overall, 61.9% (*n* = 13) of them with severe malaria were infected with Plasmodium falciparum and remainder had P. vivax infection (Table 2).

### Association between history of COVID-19 infection and Severe Malaria

Table 1 shows the association between the history of COVID-19 infection and the development of severe malaria and severe dengue among malaria and dengue patients respectively. In total, 85.7% (*n* = 12) of the participants with a history of COVID-19 infection developed severe malaria as compared to 16.1% (*n* = 9) without a history of COVID-19 infection and this difference was found to be statistically significant (*p* < 0.001).

Among individuals with severe malaria, 85.7% (*n* = 12) had a history of COVID-19 infection, compared to 16.1% (*n* = 9) of individuals without severe malaria (controls) who had a history of COVID-19 infection. This difference was found to be statistically significant (*p* < 0.001).

### Association between history of COVID-19 infection and Severe Dengue

Among individuals with severe dengue, 98.1% (*n* = 52) reported a history of COVID-19 infection, while 16.5% (*n* = 28) of individuals without severe dengue (controls) reported a history of COVID-19 infection. This difference was found to be statistically significant (*p* < 0.001).

### Factors associated with Severe malaria

Multivariable analysis of the factors associated with severe malaria is presented in Table 4. Age group of > 30 years (*p* = 0.714), males (*p* = 0.099), education below high school (*p* = 0.264) and those engaged in unskilled & semiskilled occupations (*p* = 0.665), participants with comorbidities (*p* = 0.063) and those who received 3 doses of the vaccine (*p* = 0.710) had higher chances of developing severe malaria. However, these associations were not statistically significant.

**Table 4:** Multivariable analysis of the factors associated with severity of Malaria (*n*=70)

**Characteristics**		**Crude OR (COR) (95% CI)**	***p*-value**	**Adjusted OR (aOR (95% CI)**	***p*-value**
Age(years)	<30	-------	0.404	-------	0.714
	>30	1.5 (0.549-4.430)		1.38 (0.24 - 7.73)	
Gender	Male	1.7 (0.540-5.851)	0.344	4.84 (0.74-31.64)	0.099
	Female	-------		-------	
Occupation	Skilled & Professionals	-------	0.864	-------	0.665
	Others	1.11 (0.35-3.4)		1.46 (0.259 - 8.313)	
Education	Below high school	1.08 (0.32-3.61)	0.901	3.84 (0.365-40.0)	0.264
	High school & above	-------		-------	
Socio economic status	I & II	-------	0.148	-------	0.036*
	III & below	2.0 (0.76-6.17)		7.69 (1.13-50.0)	
Comorbidities	Yes	3.9 (0.60-25.40)	0.152	23.1 (0.84-633.33)	0.063
	No	-------		-------	
History of COVID-19 infection	Yes	15.0 (3.93-57.22)	<0.001*	55.93 (7.17–435.92)	<0.001*
	No	-------		-------	
No. of doses of COVID-19 vaccine taken	2	-------	0.630	-------	0.710
	3	1.29 (0.45-3.66)		1.40 (0.23-8.40)	

*p-value significant at 0.05, -------Ref category, Cox & Snell R^2^=0.362, Nagelkerke R^2^=0.513

It was noted that participants belonging to socio-economic status classes III & below (aOR: 7.69 (95% CI: 1.13–50.0), *p* = 0.036) and those with a history of COVID-19 infection in the past (aOR: 55.93 (95% CI: 7.17–435.92), *p* < 0.001) had higher chances of developing severe malaria. In this regression model, socio-economic status and previous history of COVID-19 infection were found to be statistically significant (*p* < 0.05). However, the wide confidence intervals observed, especially for history of COVID-19 infection could be due to imbalance in the data distribution and relatively low number of severe malaria cases within the group.

The goodness of fit showed that the model fitted well (χ^2^ = 3.876, *p* = 0.868). Also, Cox & Snell R^2^ = 0.362 and Nagelkerke R^2^ = 0.513 indicate moderate predictive power of the model.

### Factors associated with Severe dengue

The multivariable analysis, presented in Table 5, depicts the factors associated with severe dengue. It revealed that individuals aged over 30 years (*p* = 0.962), males (*p* = 0.411), education below high school (*p* = 0.050), participants with comorbidities (*p* = 0.889), and participants who had received 3 doses of vaccine (*p* = 0.777) had higher chances of developing severe dengue. However, these factors were not found to be statistically significant.

**Table 5:** Multivariable analysis of the factors associated with severity of dengue (*n*=223)

**Characteristics**		**Crude OR (COR) (95% CI)**	***p*-value**	**Adjusted OR (aOR) (95% CI)**	***p*-value**
Age(years)	<30	-------	0.515	-------	0.962
	>30	1.2 (0.69-2.32)		1.0 (0.36- 2.85)	
Gender	Male	1.4 (0.79-2.531)	0.240	1.5 (0.58 - 3.70)	0.411
	Female	-------		-------	
Occupation	Skilled & Professionals	-------	<0.001*	-------	0.005*
	Others	3.5 (1.92-6.45)		6.2 (1.75 - 25.00)	
Education	Below high school	1.6 (0.83-3.10)	0.156	3.4 (1.01 - 11.29)	0.050*
	High school & above	-------		-------	
Socio economic status	I & II	-------	<0.001*	-------	<0.001*
	III & below	3.57 (1.91-6.45)		4.5 (1.85–11.11)	
Comorbidities	Yes	1.2 (0.63-2.38)	0.546	1.1 (3.84–4.01)	0.889
	No	-------		-------	
History of COVID-19 infection	Yes	250 (34.48- 1000)	<0.001*	263.7 (34.9–1987.4)	<0.001*
	No	-------		-------	
No. of doses of COVID-19 vaccine taken	2	-------	0.590	-------	0.777
	3	1.2 (0.65-2.09)		1.15 (0.44-3.04)	

*p-value significant at 0.05, ------ Reference category, Cox & Snell R^2^=0.501, Nagelkerke R^2^=0.688

It was noted that participants engaged in semiskilled & unskilled occupations (aOR: 6.2; 95% CI: 1.75-25.00; *p* < 0.001), individuals belonging to socio-economic status classes I & II (aOR: 4.5; 95% CI: 1.85–11.11; *p* < 0.001) and those with a history of COVID-19 infection (aOR: 263.7; 95% CI: 34.9-1987.4; *p* < 0.001) had higher chances of developing severe dengue. In this regression model, occupation, socio-economic status, and past COVID-19 infection emerged as statistically significant exposures associated with severe dengue (*p* < 0.05). The wide confidence intervals observed could be due to the imbalances in the data distribution within groups. However, the model demonstrated a good fit (χ2 = 11.869, *p* = 0.157), and the Cox & Snell R^2^ = 0.501 and Nagelkerke R^2^ = 0.688 values suggest a moderate predictive power of the model.

## Discussion

The study area has been endemic to vector-borne diseases for several years [11]. Both diseases, included in this study, i.e., malaria and dengue, have the potential to become fatal in many situations. Currently, the world is in the post-COVID-19 pandemic era. However, future global outbreaks of a similar nature and magnitude cannot be ruled out. Infection with COVID-19 has already been found to cause long-term immunological alterations in the human body [14]. A study by R. Ramakrishnan et al. revealed that being infected with the COVID-19 virus can cause immune exhaustion, post-viral autoimmunity, dysregulated immune metabolism, and microbial dysbiosis, contributing to the post-acute effects of COVID-19 [14]. Research has also indicated a possibility of cross-immunity between certain pathogens, suggesting that exposure to one might protect against another during a future infection. For instance, prior infection with malaria has been linked to reduced susceptibility to chikungunya [15–17]. Thus, there is a need for robust evidence on the impact of past COVID-19 infection on the severity of diseases like malaria and dengue. However, as noted, there is a lack of literature on how prior COVID-19 infection can affect the severity of these diseases.

Although the causative agents for dengue and malaria are different, both infections are transmitted through the bite of infected mosquitoes. Hence, the environment and host factors of both diseases are similar. Also, the pathogenesis of COVID-19 is remarkably similar to that of malaria and dengue, especially concerning the role of inflammation. In COVID-19, the virus triggers a strong inflammatory response, only to progress, in severe cases, to a cytokine storm. They continue to release inflammatory mediators, which not only harm different organs but also damage the vascular endothelium, resulting in endothelial dysfunction and structural damage. This endothelial injury further leads to hematologic derangements and plasma leakage, defining the patient’s clinical course. The convergence of these pathways- systemic inflammation followed by endothelial cell compromise and plasma leakage is also a key aspect of pathophysiology underpinning severe malaria and dengue. Furthermore, similarities have been identified in the interaction with host cells, specifically using the ACE2 receptor by the malaria parasite and the COVID-19 virus. Genetic variations in ACE1 and ACE2 have also been associated with malaria resistance, potentially impacting susceptibility to COVID-19 [18]. 

The present study found a significant association between a history of COVID-19 infection and the severity of dengue and malaria infections. To this effect, our study is the first to our knowledge to descriptively evaluate this association. Out of the total participants in our study, 22.9% (*n* = 67) reported having prior COVID-19 infections, which were mild and never progressed to severe disease.

Dengue fever can progress to dengue haemorrhagic fever and dengue shock syndrome if not monitored and managed promptly [19]. Although vaccines and newer drugs are developed and being tested for dengue infection, supportive care remains the mainstay of management in many countries [20]. Dengue virus exists in four different forms (serotypes 1–4), increasing the likelihood of repeated infections throughout a person’s life [21]. The Case fatality of dengue can vary from < 1–15%. Similarly, malaria is a potentially fatal infection with high morbidity and mortality if it reaches the stage of severe malaria. The progression of these diseases to their severe form can be different due to various factors like increasing age, gender, type of occupation and access to quality health care [22, 23]. In the current study, we focused on the severe cases at the time of data collection. Progression from mild or moderate disease to severe disease (malaria and dengue) was not captured.

The findings of our study suggest immunological interaction between the two diseases, although the route of entry and pathogenesis are different for both. Although these studies suggest similarities in the immunological reactions of the two pathogens, the exact sequelae and consequences of this interaction remain unclear, and hence, it is necessary to identify the factors that can influence the severity of vector-borne diseases, especially in endemic regions.

A review article by Harapan H. et al. in Indonesia to describe the impact of COVID-19 on dengue infection in dengue-endemic countries concluded that due to the identical early-stage symptoms and test characteristics of both illnesses, accurate diagnosis and therapy are made difficult. Moreover, some reports have shown cross-reactivity between serology testing for DENV and COVID-19 virus antibodies [24]. 

A study done by Wang et al. found that age, gender, socioeconomic status, occupation, and geographical location are factors that can affect the severity of malaria. They found that children, pregnant women, and the elderly are more prone to severe malaria [25]. However, our study included only adults, and it was found that those aged more than 30 years were more at risk of severe malaria infection, even though there was no statistical significance. Their finding (Wang et al.) that males have higher chances of developing severe malaria was similar to our study finding, where 72.7% of the males with malaria developed complications.

A systematic review done by Abraham Degarege et al. in 2021 reiterated that lack of education, low socio-economic status, which in turn can lead to poor housing and living conditions, are factors which increase the risk of severe malaria. This is in line with our study findings in which those aged > 30 years, males, those engaged in unskilled occupations, had education below high school and lower socio-economic classes were more at risk of developing severe malarial disease.

Socio-demographic characteristics of the participants like age, education, education, occupation, and socioeconomic status, can also determine the impact of the disease as reported by M. Rajesh Kumar Rao et al. in their study, which was carried out in the year 2018 [26]. In our study, those participants aged > 30 years had higher chances of developing severe dengue infection compared to those aged < 30 years. However, the risk was found to be more among those with a history of COVID-19 infection in the past. Also, males had more chances of developing severe malaria as compared to females, and the risk increased in participants with a history of COVID-19 infection. It was found from our study that those participants who received 3 doses of the COVID-19 vaccine had a higher chance of developing severe malaria as compared to those who received 2 doses of the vaccine. This may be due to the immune response following vaccination, which is similar to the response after an infection [26, 27]. 

The findings from this study could be used to follow up on those with a history of COVID-19 and assess the possibility of severe malaria or severe dengue in them. Also, when a patient with malaria/dengue is asked for a history of COVID-19 infection and vaccination as a routine practice so that progression to severe illness can be anticipated and close monitoring can be done as precautionary measure. Based on this, the policymakers can plan and implement additional precautionary measures to prevent severe malaria/dengue infection especially in endemic regions.

Although, in this study, an association was evaluated and established; however there is a need for further immunohistochemical and logitudinal studies to establish and strengthen biological plausibility. Thus, it will help in devising and revising health policies towards preventive measures towards malaria and dengue. In the backdrop of uncertainties, such evidence will help to gear up the preventive measures to malaria and dengue.

One of the study’s main limitations was that none of the participants reported a history of severe COVID-19 infection. There is also a possibility of recall bias among them about the history of COVID-19. All the participants were vaccinated against COVID-19, which can also mimic a similar immune response. Also, vaccination could mask the immunological pathway, and it could lead to indistinguishability to a certain extent. We used a sequential sampling technique in this study, considering the feasibility aspects and changing trend of the vector-borne disease burden. This could bring about some element of selection bias and generalizability issues. In addition, this study could not assess the causality and immunological markers due to feasibility and operational reasons.

## Conclusions

Individuals with a prior COVID-19 infection were found to have a higher occurrence of severe malaria and dengue in this study compared to those without a history of COVID-19. Furthermore, prior infection with COVID-19 appeared to worsen the seriousness of these illnesses, especially in people with additional risk factors like socioeconomic status, education level and occupation. Also, age and gender, although not statistically significant were found to increase the chances of developing severe illness. However, further longitudinal studies have to be done to confirm these findings.

## Supplementary Information

Supplementary Material 1. Case definitions for severity of malaria and dengue

Supplementary Material 2. STROBE checklist

## Data Availability

Data related to this study is available at the Open Science Framework from 10.17605/OSF.IO/MKYWC.

## Abbreviations

aOR: Adjusted Odds Ratio
CI: Confidence Interval
COVID-19: Coronavirus Disease 19
CVA: Cerebrovascular Accident
DVBDCP: District Vector Borne Disease Control Programme
HTN: Hypertension
IEC: Institutional Ethics Committee
NS1: Non-Structural Antigen 1
NVBDCP: National Vector Borne Disease Control Programme
SD: Standard Deviation
SPSS: Statistical Package for Social Sciences
T2DM: Type 2 Diabetes Mellitus
WHO: World Health Organisation
