# Supplementary material for: COVID-19 infection and its association with severe malaria & dengue: an epidemiological study from Southern India
Source: BMC Infect Dis. 2025 Jul 19;25:927. doi: 10.1186/s12879-025-11324-4 (PMC12275358; doi:10.1186/s12879-025-11324-4)
Supplement: Supplementary file 1 — Supplementary Material 1. Case definitions for severity of malaria and dengue [file 12879_2025_11324_MOESM1_ESM.docx]

**SUPPLEMENTARY MATERIAL**

**OPERATIONAL DEFINITIONS:**

1. **Severe Malaria:**

Patients diagnosed with malaria by RDT/ Microscopy having any of these features will be considered as having severe malaria:

1. Impaired consciousness/ coma
2. Repeated generalized convulsions
3. Renal failure (serum creatinine>3mg/dl)
4. Jaundice (serum bilirubin > 3mg/dl)
5. Severa anemia (Hb <5g/dl)
6. Pulmonary oedema/ acute respiratory distress syndrome
7. Hypoglycemia (plasma glucose < 40mg/dl)
8. Metabolic acidosis
9. Circulatory collapse / shock (systolic BP< 80mmhg, < 50mmhg in children)
10. Abnormal bleeding and DIC
11. Hemoglobinuria
12. Hyperthermia
13. Hyper parasitemia

2. **Mild to moderate or Uncomplicated Malaria**: Patients diagnosed with Malaria by RDT/ Microscopy and not having any of the features of severe malaria after 3 days of diagnosis were considered as mild to moderate or uncomplicated malaria.

3.**Severe dengue/ Dengue Hemorrhagic fever**: May be defined by one or more of the following:

Plasma leakage leading to shock (dengue shock) and/or fluid accumulation.

1. Severe bleeding
2. Platelet count < 100,000 cells/mm^3^
3. Hemorrhagic manifestations shown by any of the following- positive tourniquet test, petechiae, ecchymoses or purpura, or bleeding from mucosa, GI tract, injection sites or other locations.
4. Signs of shock

4. **Non-severe or Classical Dengue fever**: Patients diagnosed with Dengue and are not having any features of severe dengue infection (as listed above) after 3 days of diagnosis will be considered as non-severe or uncomplicated dengue cases.

5**. Severe COVID-19**: Patients diagnosed with COVID-19 by Rapid Antigen test (RAT) or RTPCR who required hospitalisation with invasive or non-invasive mechanical ventilation during the course of infection.

6. **Moderate COVID-19:** Patients diagnosed with COVID-19 by RAT or RTPCR who had symptoms of pneumonia but no signs of severe disease, required hospitalisation and Oxygen supply with nasal cannula or Oxygen face mask.

7.**Mild COVID-19**: Patients diagnosed with COVID-19 who are asymptomatic or having fever and uncomplicated upper respiratory tract infection without dyspnoea or hypoxemia.
